# MS-275 synergistically enhances the growth inhibitory effects of RAMBA VN/66-1 in hormone-insensitive PC-3 prostate cancer cells and tumours

**DOI:** 10.1038/sj.bjc.6604295

**Published:** 2008-03-18

**Authors:** A Khandelwal, L K Gediya, V C O Njar

**Affiliations:** 1 Department of Pharmacology and Experimental Therapeutics, University of Maryland School of Medicine, 655 West Baltimore Street, Baltimore, MD 21201-1559, USA; 2 Marlene and Stewart Greenebaum Cancer Center, University of Maryland School of Medicine, Baltimore, MD 21201-1559, USA

**Keywords:** prostate cancer (PCa), retinoic acid metabolism-blocking agents (RAMBAs), histone deacetylase inhibitors (HDACIs), VN/66-1, MS-275, combination therapy

## Abstract

Combining drugs, which target different signalling pathways, often decreases adverse side effects while increasing the efficacy of treatment. The objective of our study was to determine if the combination of our novel atypical retinoic acid metabolism-blocking agent (RAMBA) VN/66-1 and a promising histone deacetylase inhibitor *N*-(2-aminophenyl)4-[*N*-(pyridine-3-yl-methoxy-carbonyl)aminomethyl]benzamide (MS-275) would show enhanced antineoplastic activity on human PC-3 prostate cancer cells/tumours and also to decipher the molecular mechanisms of action. The combination of VN/66-1+MS-275 was found to be synergistic in inhibiting PC-3 cell growth, caused cell cytostaticity/cytotoxicity and induced marked G2/M phase arrest and apoptosis. In mice with well-established PC-3 tumours, VN/66-1 (5 and 10 mg kg^−1^ day^−1^) caused significant suppression of tumour growth compared with mice receiving vehicle alone. Furthermore, treatment with VN/66-1 (10 mg kg^−1^ day^−1^)+MS-275 (2.5 mg kg^−1^ day^−1^) for 18 days resulted in an 85% reduction in final mean tumour volume compared with control and was more effective than either agent alone. Mechanistic studies indicated that treatment of PC-3 cells/tumours with VN/66-1+MS-275 caused DNA damage (upregulation of *γ*H2AX), hyperacetylation of histones H3 and H4, upregulation of retinoic acid receptor-*β*, p21^WAF1/CIP1^, E-cadherin, and Bad and downregulation of Bcl-2. These data suggest that the mechanism of action of the combination of agents is DNA damage-induced p21 activation, resulting in inhibition of the Cdc2/cyclin B complex and accumulation of cells in G2/M phase. In addition, the combination caused modulation and induction of apoptosis. These results suggest that VN/66-1 or its combination with MS-275 may be a novel therapy for the treatment of prostate carcinoma.

Prostate cancer (PCa) is the most frequently diagnosed cancer in American men with an estimated 218 890 new cases of the disease in 2007 (Jemal *et al*, 2007). Since PCa is one of the leading causes of deaths worldwide, there is an urgent need to identify effective agents against this disease. Retinoids, rexinoids, retinoid-related molecules (RRMs) and histone deacetylase inhibitors (HDACIs) have shown promising biological activities as single agents in several preclinical studies of both haematological and solid malignancies (Altucci and Gronemeyer, 2001; Lindemann *et al*, 2004; Bolden *et al*, 2006; Njar *et al*, 2006a). Because of the heterogeneous nature of PCa, we hypothesise that agents/combination of agents affecting various pathways involved in PCa growth and survival may be advantageous.

It is well noted that ATRA (all-*trans* retinoic acid), the most active metabolite of vitamin A is required for the appropriate differentiation of normal human prostate epithelial cells (Sonneveld *et al*, 1998). All-*trans* retinoic acid undergoes cytochrome P450 metabolism to form inactive polar metabolites (Njar *et al*, 2006b). The metabolism of ATRA by CYP enzymes (particularly by CYP26) and dysregulation of gene expression contribute to the cancerous phenotype. Treatment of disorders with ATRA often leads to the development of resistance and is toxic (Freemantle *et al*, 2003).

In attempts to overcome retinoid resistance in cancer therapy (Freemantle *et al*, 2003), our group and others have developed several retinoic acid metabolism-blocking agents (RAMBAs) that are currently being investigated in several pre-clinical models of a variety of cancers and dermatological diseases (Njar *et al*, 2006b). Our RAMBAs, however, based on the retinoid scaffold are considered ‘atypical RAMBAs’, because they are also endowed with multiple anticancer activities. On the basis of several studies in our lab, it is now evident that the anticancer activities of our RAMBAs cannot be explained by inhibition of ATRA metabolism alone (Patel *et al*, 2004, 2007b; Gediya *et al*, 2005; Belosay *et al*, 2006; Huynh *et al*, 2006). We recently examined the growth inhibitory effects of one of our lead atypical RAMBAs VN/66-1 (Figure 1A) in combination with clinically used suberoylanilide hydroxamic acid (SAHA, a HDACI) on the androgen receptor-positive human prostate LNCaP cell line (Gediya *et al*, 2005). An additive growth inhibitory effect was observed. When tested against other potent RAMBAs and ATRA, VN/66-1 was found to possess the most favourable pharmacokinetic properties in mice and was relatively non-toxic (Patel *et al*, 2007a). This promising compound may also be effective in hormone-refractory PCa models.

Histone acetyltransferase and histone deacetylase (HDAC) have opposing effect on transcription (Kuo and Allis, 1998; Ito and Adcock, 2002). Often, DNA methylation and histone deacetylation of tumour suppressor genes occur in many human cancers, leading to suppression of function of these genes thereby conferring a growth advantage for the tumour cells (Robertson and Jones, 2000; Macaluso and Giordano, 2004). Indeed, it has been demonstrated that the expression and activity of HDAC1 are upregulated (two- to four-fold) in PCa compared to benign prostatic hyperplasia (Patra *et al*, 2001). Histone deacetylase inhibitors, such as SAHA, and *N*-(2-aminophenyl)4-[*N*-(pyridine-3-yl-methoxy-carbonyl)aminomethyl] benzamide (MS-275) can directly interact with the HDAC enzymes at the catalytic site and inhibit their function (Marks *et al*, 2000; Insinga *et al*, 2005; Bolden *et al*, 2006). This leads to acetylation of histones, which opens up the chromatin structure allowing transcription of antigrowth and proapoptotic genes to occur. It should be noted that MS-275 is now in phase I/II clinical trials for various solid tumours and haematological malignancies (Hess-Stumpp *et al*, 2007).

Histone deacetylase inhibitors have been reported to have growth inhibitory activities in PCa models both *in vitro* and *in vivo* (Butler *et al*, 2000; Nakayama *et al*, 2001; Gediya *et al*, 2005). In addition, HDACIs when combined with retinoids (e.g., ATRA, 9-*cis* retinoic acid (9-CRA) or 13-*cis*-retinoic acid (13-CRA)) display enhanced (additive) anticancer activities (Pili *et al*, 2001; Gediya *et al*, 2005; Qian *et al*, 2005, 2007). However, combination antiproliferative studies with the well-known synthetic retinoid, *N*-(4-hydroxyphenyl)retinamide, (4-HPR) have produced conflicting results. Gu *et al* (2006) clearly demonstrated enhanced cytotoxic effects with the combination of 4-HPR or other retinoids and HDACIs, whereas Kuefer *et al* (2007) reported that sodium butyrate and 4-HPR administered together antagonise the effects of each other. Nevertheless, these studies suggest that combinations that include retinoids and HDACIs may have a greater therapeutic action than treatment with either agent alone.

It is well established that combining drugs, which target different signalling pathways, can lessen adverse side effects while increasing the efficacy of treatment (Bevins and Zimmer, 2005; Verheul *et al*, 2007). The objective of our study was to determine if the combination of VN/66-1 and MS-275 would show enhanced (additive or synergistic) antineoplastic activity on hormone-insensitive human PC-3 PCa cells and to decipher the molecular mechanisms of action. In the present report, we describe a synergistic inhibitory effect of MS-275 and our atypical RAMBA VN/66-1 on the growth of PC-3 cells, and we also describe a synergistic inhibitory effect of MS-275+VN/66-1 administration on the growth of well-established PC-3 tumours in a xenograft model. The antiproliferative effects of these agents alone and in combination are due to DNA damage-induced modulation of cell cycle regulatory proteins including p21^WAF1/CIP1^ resulting in G2/M cell cycle arrest and induction of apoptosis via the mitochondrial pathway. Furthermore, we show that these agents, alone or in combination, exert their effect on cell growth via retinoid receptor-independent mechanisms. The novelty of this work arises from that fact that it is the first mechanistic report of a RAMBA in combination with an HDACI as a therapeutic for any disorder including cancer.

## MATERIALS AND METHODS

### Chemicals and reagents

ATRA was purchased from LKT Laboratories Inc. (St Paul, MN, USA), while VN/66-1 and MS-275 were synthesised in our laboratory. AGN193109 was kindly provided by Dr Dianne Soprano, Temple University, Philadelphia, PA, USA.

### Cell culture

The PC-3 (hormone-insensitive PCa) cell line was obtained from American Type Culture Collection (Rockville, MD, USA). C-81 cells were obtained from the lab of Dr Anne Hamburger and PC3-AR cells from the lab of Dr Yun Qiu both at the University of Maryland School of Medicine. Cells were maintained in RPMI 1640 medium supplemented with 10% fetal bovine serum (FBS) (Atlanta Biologicals, Lawrenceville, GA, USA) and 1% penicillin/streptomycin. Cells were grown as a monolayer in T75 or T150 tissue culture flasks in a humidified incubator (5% CO_2_, 95% air) at 37°C.

### Cell growth inhibition (MTT colorimetric assay)

PC-3 cells were seeded in 24- or 96-well plates (Corning Costar) at a density of 2 × 10^4^ cells per well (24 wells) or 2 × 10^3^ cells per well (96 wells). Cells were allowed to adhere to the plate for 24 h and then treated with various concentrations of VN/66-1 or MS-275 dissolved in 95% EtOH and 10% DMSO/90% EtOH, respectively. Cells were treated for 5 days with renewal of test compound and media on day 3. On the fifth day, medium was renewed and MTT (3-(4,5-dimethylthiazol-2-yl)-2,5-diphenyl-2H-tetrazolium bromide) (Sigma, St Louis, MO, USA) solution (0.5 mg MTT per ml of media) was added to the medium such that the ratio of MTT : medium was 1 : 10. The cells were incubated with MTT for 2.5 h. The medium was then aspirated and DMSO was added to solubilise the violet MTT-formazan product. The absorbance at 560 nm was measured by spectrophotometry (Victor 1420 multilabel counter, Wallac, Perkin Elmer, Waltham, MA, USA). For sequential treatment experiments, cells were treated with the first agent for 48 h, washed and treated with the second agent for another 48 h after which the assay was carried out as described above. Triplicates were carried out for each concentration and experiments were independently repeated a minimum of three times. Results are expressed as percentage of viable cells in the vehicle-treated control wells. IC_50_ values were calculated by nonlinear regression analysis using GraphPad Prism 4.0 (GraphPad Software, San Diego, CA, USA).

### Assessment of synergism, additivity or antagonism

The level of interaction (synergistic, additive or antagonistic) between VN/66-1 and MS-275 was evaluated as described previously (Chou *et al*, 1993). Synergism, additivity or antagonism was quantified by determining the combination index (CI), where CI<1, CI=1, and CI>1 indicate a synergistic, additive or antagonistic effect, respectively. Based on the classic isobologram, the CI was calculated by using the following equation: 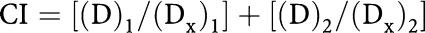 At the 75% inhibition level, (D_x_)_1_ and (D_x_)_2_ are the concentrations of VN/66-1 and MS-275, respectively, which induce a 75% inhibition in cell growth. (D)_1_ and (D)_2_ are the concentrations of VN/66-1 and MS-275, which also inhibit cell growth by 75% (as compared with single agents alone).

### Cell cycle analysis

Cells were plated in T-75 flasks containing complete RPMI 1640 medium for 24 h. Cells were serum starved after washing with phosphate-buffered saline (PBS) and incubating with RPMI 1640 (minus phenol red) with 0.2% FBS for 48 h. Under these conditions, cells were arrested in the G0/G1 phase as determined by flow cytometry. Cells were then stimulated by the addition of complete RMPI 1640 medium containing 10% FBS. Cells were treated with VN/66-1 or MS-275 for various times, washed with PBS, trypsinised, resuspended in 10 ml PBS and counted. They were then centrifuged (10 min, 2500 r.p.m. at 4°C), resuspended in PBS, fixed in 70% ice-cold ethanol and stored in −20°C until staining. Cells were stained for at least 1 h in the dark with a solution containing 20 *μ*g ml^−1^ propidium iodide (Sigma), 0.02 *μ*g ml^−1^ RNAse and 1 % Triton-X 100 (Sigma). The DNA content in the treated and mock-treated groups was measured by flow cytometry analysis using a FACSort flow cytometer (Becton Dickinson, San Jose, CA, USA); 15 000 events were analysed for each sample. ModFit LT version 3.1 (Verity Software House Inc., Topsham, ME, USA) was used to analyse cell cycle distribution.

### Flowcytometry for apoptosis detection

Cells were grown in T-75 flasks and treated with various concentrations of VN/66-1 and MS-275 for different times. Growth media were collected; cells were typsinzed, pelleted and washed with PBS. The Annexin V-FITC Apoptosis Detection Kit I was used to determine the number of early and late apoptotic/necrotic cells (BD Biosciences, San Jose, CA, USA). Cells were analysed using a FACSort flow cytometer as mentioned above; 10 000 events were analysed. CellQuest Pro version 5.2.1 (BD Biosciences) was used to analyse the distribution of cells.

### Immunofluorescence

PC-3 cells were grown on eight-chamber glass slides and treated with various agents for 24 h. Immunofluorescence staining was carried out as described previously (Phatak *et al*, 2007). After fixing with methanol : acetone (1 : 1), cells were stained with *γ*-H_2_AX (Upstate, Waltham, MA, USA) and counterstained with 4′,6-diamidino-2-phenylindole dihydrochloride (DAPI) (Sigma). Cells were viewed using a Zeiss Axiovert Fluorescent Microscope (Carl Zeiss, Gottingen, Germany). Images were processed using Openlab Volume Deconvolution software for Mac (Improvision, Lexington, MA, USA).

### Quantitative real-time PCR

PC-3 cells were grown in T-75 flasks and were treated with various agents for 24 h. Cells were then harvested and RNA isolated using the RNeasy Mini Kit (Qiagen Inc., Valencia, CA, USA). cDNA was synthesised using the ReactionReady First Strand cDNA Synthesis Kit (SuperArray Bioscience Corporation, Frederick, MD, USA) as directed by the manufacturer. The RT^2^ Real-Time SYBR Green PCR Master Mix and primers (retinoic acid receptor (RAR)*β*2, cyclin D1 and *β*-actin) were also obtained from SuperArray. qRT–PCR was performed on a ABI 7900HT machine (Applied Biosystems, Foster City, CA, USA). The relative levels of RAR*β*2 and cyclin D1 expression were determined with reference to the internal *β*-actin control by calculating 2^−ΔΔCT^.

### Tumour xenograft studies

All animal studies were performed according to the guidelines approved by the Institution of Animal Care and Use Committee (IACUC) of the University of Maryland School of Medicine. Male SCID mice 4–6 weeks of age (weighing 20–25 g) were obtained from the National Cancer Institute (Frederick, MD, USA). Mice were maintained in a controlled environment of light, humidity and temperature and were given food and water *ad libitum*. Subconfluent PC-3 cells were detached using citric saline, suspended in PBS, centrifuged and resuspended in Matrigel (BD Biosciences) (10 mg ml^−1^) at 5.0 × 10^7^ cells per ml. Tumour cells were inoculated subcutaneously into each flank of the mice. Drug treatment was initiated when tumours were palpable (∼75 mm^3^). VN/66-1 and MS-275 were suspended in 0.3% hydroxypropyl cellulose in saline. VN/66-1 (10 mg kg^−1^) and MS-275 (2.5 mg kg^−1^) were administered daily (5 days per week) by subcutaneous injection. Tumour measurements were taken twice a week with a digital caliper and mice were weighed once a week. Tumour volume was calculated by the formula (4/3 *πr*_1_^2^ × *r*_2_) with *r*_1_ and *r*_2_ being the larger and smaller radii, respectively.

### Western blot analysis

For immunoblot detection of various proteins, PC-3 cells were cultured as described above in T75 flasks. Cells were treated with VN/66-1 or MS-275 and whole cell lysates were prepared using lysis buffer containing 0.1 M Tris, 0.5% Triton X-100 and protease inhibitor. For detection of *γ*-H2AX and Acetyl H3 and H4 (Upstate, Lake Placid, NY, USA), histones were extracted as described previously (Phatak *et al*, 2007). Protein content was determined using the Bradford Assay (Bio-Rad, Hercules, CA, USA). Protein was subjected to SDS–PAGE and transferred onto nitrocellulose membrane. Antibodies against PARP, RAR*β* and E-cadherin were purchased from Santa Cruz Biotechnology (Santa Cruz, CA, USA). Antibodies against Bax, Bad, Bcl-2, cdc25C, p-cdc2 (Thr 14) and p-cdc2 (Tyr 15), and *β*-actin were purchased from Cell Signaling (Danvers, MA, USA) and p21 was purchased from Upstate (Millipore, Billerica, MA, USA). Membranes were then incubated with secondary antibody (Bio-Rad) at room temperature for 1 h. Bands were visualised by chemiluminescence (Millipore). Protein expression was normalised to *β*-actin and densitometry was carried out using ImageQuant 5.0 (Molecular Dynamics, Sunnyvale, CA, USA).

### Statistical analysis

All experiments were carried out in at least triplicates and are expressed as mean±s.e. where applicable. Treatments were compared to controls using the Students’ *t*-test with either GraphPad Prism or Sigma Plot. Various treatment groups were compared using the analysis of variance (ANOVA). *P*-values less than 0.05 were considered to be statistically significant.

## RESULTS

### Effect of VN/66-1 and MS-257 alone or in combination on PC-3 cell viability

**Figure 1:**
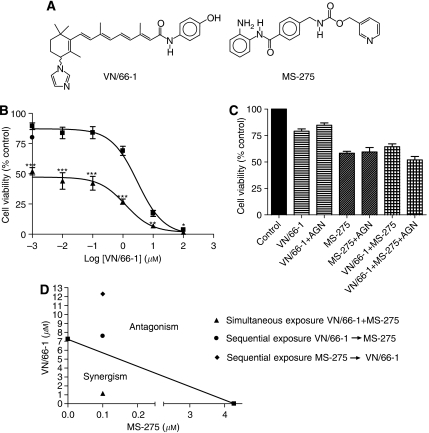
(**A**) Chemical structures of RAMBA, VN/66-1 and HDAC inhibitor, MS-275. (**B**) Dose–response curve of VN/66-1 alone and the combination of VN/66-1 and MS-275 (simultaneous treatment) in PC-3 cells. ▪ denotes various concentrations of VN/66-1 alone; ▴ denotes VN/66-1 at various concentrations plus 0.1 *μ*M of MS-275; • shows the effect of 0.1 *μ*M MS-275 alone. ^***^P<0.005; ^**^P<0.01; ^*^P<0.05 (*t*-test). (**C**) The effect of AGN193109 (abbreviated AGN), a pan-RAR antagonist on cell viability (simultaneous treatment). PC-3 cells were treated with 1 *μ*M VN/66-1 and/or 0.1 *μ*M MS-275, and 0.1 *μ*M AGN193109. ^***^P<0.0001 (*t*-test). Data are mean (±s.e.) of at least three independent experiments (**B** and **C**). (**D**) Isobologram analysis of PC-3 cell growth inhibition by VN/66-1 and MS-275 simultaneously and sequentially. The IC_75_ values of each drug are plotted on the axes; the solid line represents the additive effect, while the points represent the concentrations of each compound resulting in 75% growth inhibition. Arrow indicates sequence of treatment.

The effect of VN/66-1 and MS-275 on PC-3 cell viability was examined by the MTT assay (Figure 1B). VN/66-1 inhibited PC-3 cell viability with an IC_50_ of 2.19 *μ*M and MS-275 was found to have an IC_50_ of 0.18 *μ*M. However, in the combination treatment, there was a striking shift in the dose–response curve when compared to VN/66-1 alone (Figure 1B). This was seen after various doses (0.01–10 *μ*M) of VN/66-1 were treated with a low dose of MS-275 (0.1 *μ*M, a dose that inhibited cell viability by ∼20%, Figure 1B). *N*-(2-aminophenyl)4-[*N*-(pyridine-3-yl-methoxy-carbonyl)aminomethyl]benzamide (0.1 *μ*M) significantly enhanced the antiproliferative activity of VN/66-1 by >2000-fold (IC_50_ from 2.19 *μ*M to <1.0 nM).

Since the simultaneous treatment was found to be effective, it was important to determine whether sequential treatment would have similar effects. For the sequential treatment, cells were treated with each compound for 48 h. It was found that only the simultaneous treatment had a synergistic effect in inhibiting PC-3 cell viability as shown by a classical isobologram (Figure 1C). Synergism was further assessed by calculating the CI values. A CI<1, indicative of synergism, was only found for the simultaneous treatment (CI=0.18), while the sequential treatments were found to be antagonistic (CI=1.10 for VN/66-1 first; and CI=1.72 for MS-275 first) at the 75% inhibition level (Table 1). Furthermore, only the simultaneous treatment had a synergistic effect at all three (50, 75 and 90%) inhibition levels.

Based on these results, cells were treated at concentrations, which correspond to a 75% level of inhibition (10 *μ*M VN/66-1 and 5 *μ*M MS-275) for all subsequent experiments unless otherwise stated. Because the combination was found to be synergistic, these concentrations were halved for simultaneous treatments (5 *μ*M VN/66-1+2.5 *μ*M MS-275). This concentration of VN/66-1 is biologically relevant, because it is well below the maximum plasma concentration (26.9 μM), which was achieved in mice following a single subcutaneous administration of 10 mg kg^−1^ of VN/66-1 (Patel *et al*, 2007a). It should also be noted that these concentrations of MS-275 have been used previously for a similar study using PC-3 cells (Chen *et al*, 2005). Furthermore, it has been reported that MS-275 has an IC_50_ value of 4.8 *μ*M in inhibiting HDAC activity in nuclear extracts (Miller *et al*, 2003).

We also tested PC3-AR cells, PC-3 cells infected with the androgen receptor and C-81 cells (high passage LNCaP cells) (Igawa *et al*, 2002) to see if they responded to the same extent as the PC-3 cells. Since PC-3 cells are known to be AR-negative and clinically most prostate tumours express the AR, it was important to determine if the effects of the combination treatment were independent of AR status. We found that the IC_50_ for the combination of VN/66-1+MS-275 (various concentrations of VN/661-1+0.1 *μ*M MS-275) in the PC3-AR cells was 370 nM (dose–response curve not shown). Similar results were obtained with C-81 cells (data not shown). It is evident that the combination is effective independent of AR status of the PCa cells.

### PC-3 cell growth inhibition by VN/66-1 or MS-275 or their combination is independent of retinoid receptors

Previously we had shown that VN/66-1 does not bind to or cause transactivation of the RARs (*α*, *β* or *γ*) (Patel *et al*, 2007b). To further examine the role of the RARs in the mechanism of action of either VN/66-1 or MS-275, we tested the effect of these agents in combination with AGN193109, a pan-RAR antagonist (Agarwal *et al*, 1996). AGN193109 did not have any effect on 1 *μ*M of VN/66-1, 0.1 *μ*M of MS-275 or the combination (Figure 1D). Thus, we conclude that neither VN/66-1 nor MS-275 require the RARs to exert their growth inhibitory effects. However, this is not to say that the RARs are not involved in the mechanism of action of these agents, it only shows that the RARs are not necessary. This complements findings reported for the most active metabolite of 4-HPR, 4-oxo-fenretinide (4-oxo-4-HPR) (Villani *et al*, 2006) and the unhydrolyzable fenretinide analog, 4-hydroxybenzylretinone (Anding *et al*, 2007). It is important to note that VN/66-1 is structurally similar to these RRMs.

### Effects of VN/66-1 and MS-275 on cell cycle and proliferation

**Figure 2:**
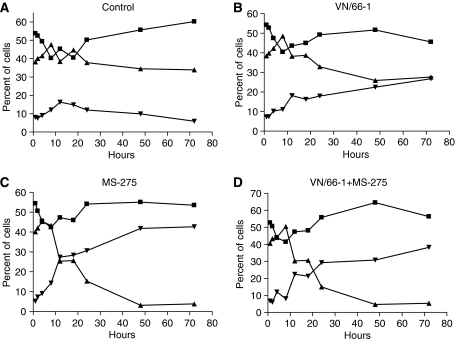
Percent of PC-3 cells in each phase of the cell cycle. (**A**) Control; (**B**) VN/66-1 (10 *μ*M); (**C**) MS-275 (5 *μ*M); (**D**) VN/66-1+MS-275 (5+2.5 *μ*M, respectively). ▪ represents the cells in the G0/G1 phase; ▴ represents cells in the S phase; ▾ represents cells in the G2/M phase of the cell cycle.

**Figure 3:**
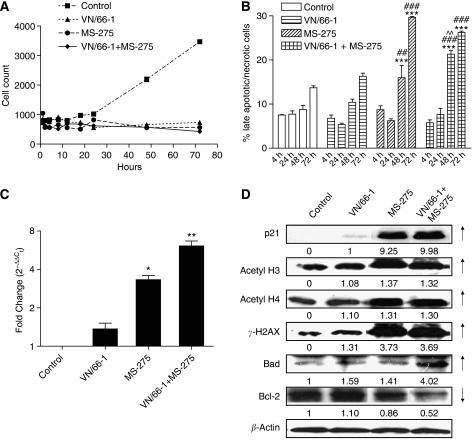
(**A**) Cell count over time after treatment of PC-3 cells. VN/66-1 (10 *μ*M), MS-275 (5 *μ*M) and the combination VN/66-1+MS-275 (5+2.5 *μ*M) were used, respectively. *P*<0.001 for 18–72 h *vs* control (ANOVA) (two-way ANOVA was carried out and most time points were found to be significantly different for different treatments, but values are not reported here). (**B**) The percent of late apoptotic/necrotic cells as determined by Annexin V-FITC and PI positive staining by flow cytometry. ^***^P<0.001 compared to control; ^###^*P*<0.001 compared to VN/66-1; ^##^*P*<0.01 compared to VN/66-1; ^^^^*P*<0.01 compared to MS-275 (ANOVA). (**C**) RAR*β* mRNA expression after treatment of PC-3 cells for 24 h. ^*^P<0.01 and ^**^P<0.001 *vs* control and VN/66-1 alone (*t*-test). Data are mean (±s.e.) of three independent experiments (**A**–**C**). (**D**) Western blot analysis after treatment of PC-3 cells for 24 h (p21, acetyl H3, H4 and *γ*-H2AX) or 4 h (Bad, Bcl-2). Blots are representative of three independent experiments. Note: the same concentrations as stated for (**A**) were used for subsequent experiments.

VN/66-1 and MS-275 alone as well as the combination were found to induce a strong G2/M phase arrest of the cell cycle when compared to vehicle-treated cells (Figure 2A–D). Previously, studies have reported that HDACIs generally induce a G1 phase arrest at lower concentrations and a G2/M phase arrest at higher concentrations (Richon *et al*, 2000; Lindemann *et al*, 2004). We confirmed that the cells were indeed not arrested in G1 by looking at cyclin D1 mRNA levels by RT–PCR, a marker for G1 phase arrest. We found that cyclin D1 mRNA levels were not significantly altered after treatment with either of the agents or the combination (data not shown). Furthermore, an increase in p21^WAF1/CIP1^ (hereafter referred to as p21), the cyclin-dependent kinase inhibitor, was observed. This is especially important since p21 has been known to be involved in the ability of HDACIs to block proliferation (Acharya *et al*, 2005). p21 protein expression was found to be upregulated (9.25-fold) with MS-275 alone and even more (10-fold) in the combination of VN/66-1 and MS-275 (Figure 3D).

Both agents alone and the combination of VN/66-1 and MS-275 were found to be cytostatic and cytotoxic. A cell count over time revealed that while vehicle treated cells continued to proliferate, cells in the three treatment groups did not (Figure 3A). Furthermore, the number of late apoptotic and necrotic cells increased over time in the three treatment groups. Apoptotic control cells remained fairly constant until 72 h at which time these cells were most likely dying from depleted serum levels in the media (Figure 3B). This indicated that the cells were actually dying after treatment with VN/66-1 or MS-275 alone and with the combination of both agents. Taken together, these data suggest that that cell cycle arrest followed by apoptosis leads to the reduced PC-3 cell survival that was observed.

### Treatments with VN/66-1 or MS-275 or their combination reactivate RAR*β*2

Retinoic acid receptor-*β*2, a known tumour suppressor gene, which has been shown to be silenced in PC-3 cells (Nakayama *et al*, 2001). *N*-(2-aminophenyl)4-[*N*-(pyridine-3-yl-methoxy-carbonyl)aminomethyl]benzamide has previously been shown to reactivate RAR*β*2 in a variety of cell lines including prostate, renal carcinoma and breast cancer cells (Hess-Stumpp *et al*, 2007). We found that while MS-275 alone induced more than a three-fold increase in RAR*β*2 expression, the combination induced almost a seven-fold increase as determined by RT–PCR (Figure 3C).

### Effect of VN/66-1 and MS-275 on PC-3 cell protein expressions

The levels of histone acetylation in PC-3 cells were determined after a 24 h treatment. Histones were isolated and western blot analysis revealed that there was an increase in acetylated histones 3 and 4 in the MS-275 and combination-treated cells (Figure 3D). Increased acetylation of H3 and H4 is especially important because cells that are RAR*β* negative (LNCaP, PC-3 and DU-145 PCa cell lines) have been shown to be hypoacetylated at H3 and H4 (Nakayama *et al*, 2001). This is another reason why combining an HDACI with a RAMBA is especially important for PCa treatment. Furthermore, deacetylation of these two histones around the promoter region has also been implicated in the silencing or inactivation of p21 (Gui *et al*, 2004; Ocker and Schneider-Stock, 2007).

A general screen to determine whether apoptosis was occurring via the mitochondrial pathway revealed that Bad levels were increased and Bcl-2 levels were decreased in the combination-treated group (Figure 3D). Furthermore, staining cells with Annexin V/FITC and PI revealed that there was a time-dependent increase in the number of early (Annexin V-FITC positive, PI negative; (data not shown)) and late (Annexin V-FITC and PI positive) apoptotic cells after treatment with VN/66-1 and/or MS-275 (Figure 3B).

### VN/66-1 in combination with MS-275 induces strong DNA damage

**Figure 4:**
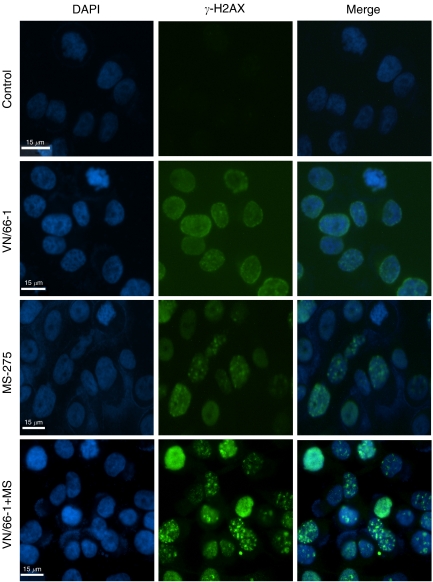
Immunocytochemistry performed with PC-3 cells after a 24 h treatment to determine the occurrence of DNA damage. Concentrations used are as follows: VN/66-1 (10 *μ*M), MS-275 (5 *μ*M) and the combination VN/66-1+MS-275 (5+2.5 *μ*M), respectively.

The phosphorylation of histone variant H2AX, *γ*-H2AX was observed by immunofluorescent staining of PC-3 cells after a 24 h treatment with the agents. *γ*-H2AX, which is a maker for DNA damage, was found to be expressed in all three treatment groups, but strong foci were only seen in the combination group (Figure 4). When *γ*-H2AX-stained cells were overlaid with DAPI-stained cells, it was apparent that the nucleus was intact and the DNA damage was occurring within the nucleus. The occurrence of DNA damage was confirmed by western blot analysis with the *γ*-H2AX antibody (Figure 3D).

### The combination of VN/66-1 and MS-275 shows an inhibitory effect on PC-3 prostate tumour growth *in vivo*

To demonstrate therapeutic activity of VN/66-1+MS-275, an *in vivo* antitumour study was conducted. PC-3 xenografts were grown in male SCID mice and treated with 10 mg kg^−1^ of VN/66-1 or 2.5 mg kg^−1^ of MS-275 or the combination of the two (subcutaneous administration). An initial dose–response study was performed to determine which doses of VN/66-1 and MS-275 would be best for the study. Three doses each of VN/66-1 (5, 10 and 20 mg kg^−1^) and MS-275 (5, 10 and 15 mg kg^−1^) were examined in a PC-3 xenograft model (data not shown). The purpose of the dose response study was to select a dose, which was not only effective in inhibiting tumour growth but also displayed minimal toxicity in the mice. Moderate weight loss was observed with the higher doses of MS-275; however, no other toxic events were noted with either compound. Based on the results that 5 mg kg^−1^ was very effective in inhibiting tumour growth, we decided to use even a lower dose for the combination study; hence, 2.5 mg kg^−1^ was administered for MS-275 alone in combination with 10 mg kg^−1^ of VN/66-1.

**Figure 5:**
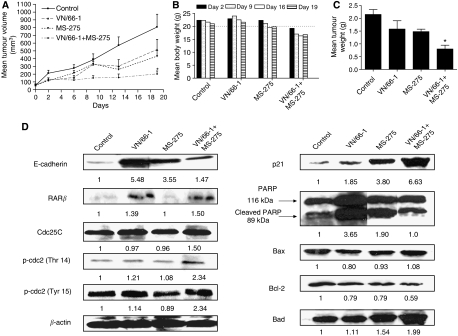
(**A**) The effect of VN/66-1 (10 mg kg^−1^), MS-275 (2.5 mg kg^−1^) and the combination (10+2.5 mg kg^−1^, respectively) in a PC-3 xenograft model in male SCID mice. Mice (*n*=5) were injected subcutaneously QD, tumours were measured twice a week. Statistical significance was determined by the *t*-test. ^*^P<0.05. Data are mean (±s.e.) (**B**) mean body weights. Mice were weighed once a week for the duration of the study. The dotted line at 20 g shows that the mice in the combination group weighed less than the mice in the other groups at the start of the study. (**C**) Mean tumour weights taken upon killing all mice and collecting tumours. ^*^P<0.05. Data are mean (±s.e.). (**D**) Western blot analysis of protein expression in PC-3 tumours taken from mice.

We found that the combination of VN/66-1 and MS-275 was the most efficacious and inhibited PC-3 tumour growth by more than 85% (Figure 5A). The mean change in tumour volume data corresponded well with the mean tumour weights for each group (Figure 5C). Furthermore, no overt weight loss was seen in any of the treatment groups (Figure 5B). Although it may appear that the combination group had lower mean body weights than the other groups, it is important to note that this group had the lowest body weights at the start of the treatment. A dotted line is drawn from 20 g on Figure 5B to clarify this. These *in vivo* results confirm our *in vitro* results that VN/66-1 and MS-275 in combination inhibit cell and tumour growth more effectively than either of the agents alone.

### Effect of treatment on PC-3 tumour protein expressions

Loss of E-cadherin, which is involved in adhesion between epithelial cells has been associated with tumour progression and metastasis (Mareel and Leroy, 2003). Thus, E-cadherin upregulation is often associated with increased differentiation of cancer cells. Immunoblot analysis with the tumour samples revealed that there was an increase in E-cadherin expression to some extent in the treatment groups (Figure 5C). Expression of p21 was also upregulated as was previously seen in whole cells. Since we had earlier demonstrated that apoptosis was taking place *in vitro*, we wanted to confirm if it was taking place *in vivo*. Thus, we examined the cleavage of PARP, and expression of Bax, Bad and Bcl-2. Although low levels of PARP cleavage was seen in the control tumours at a basal level, which is often associated with tumours, higher levels of the cleaved fragment (89 kDa) were observed in the treatment groups. Bax and Bad levels, which are both proapoptotic proteins, were elevated in the combination groups. Bcl-2, an antiapoptotic protein was found to have decreased levels in individual and combination groups (Figure 5D). These data support our proposed mechanism that apoptosis is taking place via the intrinsic pathway.

To further examine the occurrence of the G2/M phase arrest, the protein expression of various G2/M transition markers was assessed. It has been established that inhibitory phosphorylation of Cdc2 on Thr 14 and Tyr 15 causes the accumulation of inactive cyclin B1/Cdc2 complex (Mueller *et al*, 1995). Thus, we examined the expression of p-Thr 14 and p-Tyr 15 in the tumours. Both proteins showed increased levels in the combination treatment group. Cdc25C, a phosphatase that mediates the conversion of Cdc2 from an inactive to an active form was found to have unchanged levels in the tumours (Figure 5D). These data suggest that G2/M arrest is taking place in the tumours as well.

Protein expression of tumour suppressor gene, RAR*β* was found to be increased in the tumours of the VN/66-1 and combination-treated animals (Figure 5D). This is especially important because protein expression of RAR*β* could not be detected in whole cells, which is why mRNA expression was determined by a more sensitive method in these cells. Thus, it is quite significant that RAR*β* protein expression could be detected in the tumours.

## DISCUSSION

In the present study, we demonstrated, for the first time, a synergistic inhibitory effect of a RAMBA VN/66-1 and MS-275 on the growth of hormone-insensitive human PC-3 PCa cells and tumours. We also present data to suggest the mechanisms of action of these agents. A few studies have been carried out combining MS-275 and isomers of ATRA (9-CRA or 13-CRA) as recently reported in a review by Hess-Stumpp *et al* (2007). Although these two compounds have exhibited additive effects with MS-275, the therapy may be problematic for chronic use because of potential development of resistance due to induction of ATRA metabolism CYP26 enzymes (Freemantle *et al*, 2003; Njar *et al*, 2006b). In contrast, the combination of VN/66-1 and MS-275 is unlikely to succumb to this potential side effect, because VN/66-1 is a potent RAMBA and does not induce CYP26 enzymes in cancer cells (Patel *et al*, 2004; Huynh *et al*, 2006). Additionally, we have shown that while ATRA causes alopecia, skin scaling and loss of body weight at relatively low doses, VN/66-1 does not exhibit these toxic events even at doses as high as 45.7 mg kg^−1^ day^−1^ for 14 consecutive days in mice (Patel *et al*, 2007a).

In this study, we examined the combinatorial effects of VN/66-1 and MS-275 in hormone-refractory PC-3 cells. We observed that the two agents, when administered simultaneously, had a synergistic effect in inhibiting PC-3 cell growth. We also studied the effects of each sequential treatment, VN/66-1 first and then MS-275 first. Although the sequential treatments showed antagonistic effects, this is not the first time a sequential treatment with an HDACI has been found to be antagonistic, which was found to be due to conflicting signalling pathways (Kuefer *et al*, 2007). This portrays the importance of careful investigation into the effects of combination treatments and why it should not be assumed that, because two agents are effective as monotherapies, they will be effective in combination (Bevins and Zimmer, 2005; Hurtubise and Momparler, 2006).

We went on to show that both agents alone and in combination induced a G2/M phase arrest of the cell cycle in PC-3 cells. We also found that the combination was cytostatic as well as cytotoxic by blocking proliferation and inducing apoptosis. Together, these data suggest that cell cycle arrest followed by apoptosis leads to the reduced cell survival observed in the PC-3 cells. We propose that apoptosis is occurring via the mitochondrial pathway (intrinsic) based on the increased protein expression of Bad and decreased protein expression of Bcl-2. Retinoic acid receptor-*β*2, a tumour suppressor gene silenced in the PC-3 cells, was found to be reactivated and had approximately a seven-fold increase in mRNA levels in the combination-treated cells. DNA damage was also found to take place rather strongly in the VN/66-1+MS-275 group after a 24 h treatment. A marked increase in histone 3 and 4 acetylation was also observed after a 24 h treatment, as was increased protein expression of p21. Acetylation of these two histones can be directly linked to RAR*β*2 reactivation and p21 activation, since promoters of these genes have been found to be deacetylated at H3 and H4 (Lotan *et al*, 2000; Gui *et al*, 2004).

*In vivo* studies confirmed the *in vitro* results and showed that VN/66-1 (10 mg kg^−1^) in combination with MS-275 (2.5 mg kg^−1^) is very effective in suppressing PC-3 tumour growth. It is important to point out that although the combination is additive *in vivo* at the doses used, higher doses would produce a synergistic effect. Furthermore, a slightly higher dose may completely prevent further growth of the initial tumours in the combination group. Even the doses used in this study are extremely effective in inhibiting tumour growth, moreover, no overt toxicity such as alopecia, skin scaling, loss of body weight or mortality was observed in the combination group. An increase in the protein expression of Bad and decrease in the expression of Bcl-2 coupled with PARP cleavage suggests that the mechanism of apoptosis is via the intrinsic pathway. Differentiation was also observed by an increase in E-cadherin protein expression in the treatment groups. Retinoic acid receptor-*β* and p21 protein expression were also upregulated in the combination treatment tumours. G2/M transition markers were examined in tumours to confirm the G2/M arrest seen in cells.

**Figure 6:**
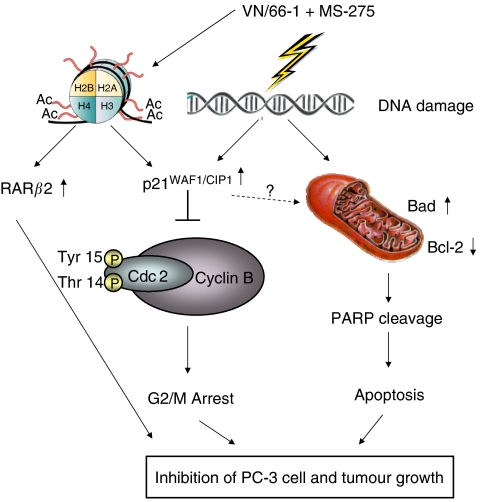
Schematic representation of the effect of VN/66-1+MS-275 on DNA damage-induced G2-M cell cycle arrest and apoptosis.

As summarised in Figure 6, the mechanism of action of the combination appears to be through the induction of DNA damage, which is known to activate p21. Activated p21 then inhibits the Cdc2/cyclin B complex, which is required for G2/M progression (Weinberg, 2007). Furthermore, it has previously been shown that p21 and p53 are necessary to maintain a G2 arrest following DNA damage (Bunz *et al*, 1998). However, it is clear in this study that p21 induction is taking place in a p53-independent manner in PC-3 cells, since it is well established that this cell line carries mutated p53 (Horoszewicz *et al*, 1980). This accounts for the G2/M arrest, which is seen in the cells. Acetylation of histones H3 and H4 also contributes to p21 activation as well as to RAR*β* activation. This cell cycle block in conjunction with apoptosis and RAR*β* activation leads to eventual inhibition of PC-3 cell and tumour growth. The wide arrays of the anticancer properties of the two agents indicate that the biological mechanisms of actions of these agents are diverse thus making them more attractive for pre-clinical and clinical development for the treatment of PCa.

In conclusion, we have found a potent combination, which is effective against hormone-refractory PCa cells and tumours. This combination of RAMBA VN/66-1 and HDACI MS-275 is highly effective in inhibiting PC-3 cell and tumour growth. In the near future, we plan to repeat the *in vivo* study with oral dosing, since MS-275 is orally bioavailable and is a preferred route of administration (Hess-Stumpp *et al*, 2007). Additionally, we propose to further investigate the mechanism of induction of DNA damage. These are the first studies to specifically explore the anticancer activities and biological mechanisms of VN/66-1 in combination with MS-275 in PCa. On the basis of these profound results, further pre-clinical studies are warranted to develop the combination of VN/66-1 and MS-275 for the treatment of PCa.

## Figures and Tables

**Table 1:** Combination index values for the three drug combinations at 50, 75 and 90% inhibition levels on PC-3 cell viability

	**CI values**		
**Drug combination**	**50%**	**75%**	**90%**
VN/66-1+MS-275	0.56	0.18	0.24
VN/66-1 → MS-275	1.85	1.10	0.69
MS-275 → VN/66-1	0.56	1.72	4.74

Arrows indicate the sequence of treatment.

## References

[bib1] Acharya MR, Sparreboom A, Venitz J, Figg WD (2005) Rational development of histone deacetylase inhibitors as anticancer agents: a review. Mol Pharmacol 68: 917–932 15955865 10.1124/mol.105.014167

[bib2] Agarwal C, Chandraratna RA, Johnson AT, Rorke EA, Eckert RL (1996) AGN193109 is a highly effective antagonist of retinoid action in human ectocervical epithelial cells. J Biol Chem 271: 12209–12212 8647816 10.1074/jbc.271.21.12209

[bib3] Altucci L, Gronemeyer H (2001) The promise of retinoids to fight against cancer. Nat Rev Cancer 1: 181–193 11902573 10.1038/35106036

[bib4] Anding AL, Chapman JS, Barnett DW, Curley Jr RW, Clagett-Dame M (2007) The unhydrolyzable fenretinide analogue 4-hydroxybenzylretinone induces the proapoptotic genes GADD153 (CHOP) and Bcl-2-binding component 3 (PUMA) and apoptosis that is caspase-dependent and independent of the retinoic acid receptor. Cancer Res 67: 6270–6277 17616685 10.1158/0008-5472.CAN-07-0727

[bib5] Belosay A, Brodie AM, Njar VC (2006) Effects of novel retinoic acid metabolism blocking agent (VN/14-1) on letrozole-insensitive breast cancer cells. Cancer Res 66: 11485–11493 17145897 10.1158/0008-5472.CAN-06-2168

[bib6] Bevins RL, Zimmer SG (2005) It's about time: scheduling alters effect of histone deacetylase inhibitors on camptothecin-treated cells. Cancer Res 65: 6957–6966 16061681 10.1158/0008-5472.CAN-05-0836

[bib7] Bolden JE, Peart MJ, Johnstone RW (2006) Anticancer activities of histone deacetylase inhibitors. Nat Rev Drug Discov 5: 769–784 16955068 10.1038/nrd2133

[bib8] Bunz F, Dutriaux A, Lengauer C, Waldman T, Zhou S, Brown JP, Sedivy JM, Kinzler KW, Vogelstein B (1998) Requirement for p53 and p21 to sustain G2 arrest after DNA damage. Science 282: 1497–1501 9822382 10.1126/science.282.5393.1497

[bib9] Butler LM, Agus DB, Scher HI, Higgins B, Rose A, Cordon-Cardo C, Thaler HT, Rifkind RA, Marks PA, Richon VM (2000) Suberoylanilide hydroxamic acid, an inhibitor of histone deacetylase, suppresses the growth of prostate cancer cells *in vitro* and *in vivo*. Cancer Res 60: 5165–5170 11016644

[bib10] Chen CS, Weng SC, Tseng PH, Lin HP, Chen CS (2005) Histone acetylation-independent effect of histone deacetylase inhibitors on Akt through the reshuffling of protein phosphatase 1 complexes. J Biol Chem 280: 38879–38887 16186112 10.1074/jbc.M505733200

[bib11] Chou TC, Tan QH, Sirotnak FM (1993) Quantitation of the synergistic interaction of edatrexate and cisplatin *in vitro*. Cancer Chemother Pharmacol 31: 259–264 8422687 10.1007/BF00685668

[bib12] Freemantle SJ, Spinella MJ, Dmitrovsky E (2003) Retinoids in cancer therapy and chemoprevention: promise meets resistance. Oncogene 22: 7305–7315 14576840 10.1038/sj.onc.1206936

[bib13] Gediya LK, Chopra P, Purushottamachar P, Maheshwari N, Njar VC (2005) A new simple and high-yield synthesis of suberoylanilide hydroxamic acid and its inhibitory effect alone or in combination with retinoids on proliferation of human prostate cancer cells. J Med Chem 48: 5047–5051 16033284 10.1021/jm058214k

[bib14] Gu J, Zhao X, Spanjaard RA, Chen TC, Flanagan JN, Boosalis M, Perrine SP, Faller DV (2006) Histone deacetylase inhibitors sensitize human prostate cancer cell lines to growth inhibitory suppression and apoptosis by retinoids. J Cancer Mol 2: 25–36

[bib15] Gui CY, Ngo L, Xu WS, Richon VM, Marks PA (2004) Histone deacetylase (HDAC) inhibitor activation of p21WAF1 involves changes in promoter-associated proteins, including HDAC1. Proc Natl Acad Sci USA 101: 1241–1246 14734806 10.1073/pnas.0307708100 PMC337037

[bib16] Hess-Stumpp H, Bracker TU, Henderson D, Politz O (2007) MS-275, a potent orally available inhibitor of histone deacetylases – the development of an anticancer agent. Int J Biochem Cell Biol 39: 1388–1405 17383217 10.1016/j.biocel.2007.02.009

[bib17] Horoszewicz JS, Leong SS, Chu TM, Wajsman ZL, Friedman M, Papsidero L, Kim U, Chai LS, Kakati S, Arya SK, Sandberg AA (1980) The LNCaP cell line – a new model for studies on human prostatic carcinoma. Prog Clin Biol Res 37: 115–132 7384082

[bib18] Hurtubise A, Momparler RL (2006) Effect of histone deacetylase inhibitor LAQ824 on antineoplastic action of 5-Aza-2′-deoxycytidine (decitabine) on human breast carcinoma cells. Cancer Chemother Pharmacol 58: 618–625 16783580 10.1007/s00280-006-0225-6

[bib19] Huynh CK, Brodie AM, Njar VC (2006) Inhibitory effects of retinoic acid metabolism blocking agents (RAMBAs) on the growth of human prostate cancer cells and LNCaP prostate tumour xenografts in SCID mice. Br J Cancer 94: 513–523 16449997 10.1038/sj.bjc.6602971 PMC2361176

[bib20] Igawa T, Lin FF, Lee MS, Karan D, Batra SK, Lin MF (2002) Establishment and characterization of androgen-independent human prostate cancer LNCaP cell model. Prostate 50: 222–235 11870800 10.1002/pros.10054

[bib21] Insinga A, Monestiroli S, Ronzoni S, Gelmetti V, Marchesi F, Viale A, Altucci L, Nervi C, Minucci S, Pelicci PG (2005) Inhibitors of histone deacetylases induce tumor-selective apoptosis through activation of the death receptor pathway. Nat Med 11: 71–76 15619634 10.1038/nm1160

[bib22] Ito K, Adcock IM (2002) Histone acetylation and histone deacetylation. Mol Biotechnol 20: 99–106 11876304 10.1385/MB:20:1:099

[bib23] Jemal A, Siegel R, Ward E, Murray T, Xu J, Thun MJ (2007) Cancer statistics, 2007. CA Cancer J Clin 57: 43–66 17237035 10.3322/canjclin.57.1.43

[bib24] Kuefer R, Genze F, Zugmaier W, Hautmann RE, Rinnab L, Gschwend JE, Angelmeier M, Estrada A, Buechele B (2007) Antagonistic effects of sodium butyrate and *N*-(4-hydroxyphenyl)-retinamide on prostate cancer. Neoplasia 9: 246–253 17401464 10.1593/neo.06766 PMC1838581

[bib25] Kuo MH, Allis CD (1998) Roles of histone acetyltransferases and deacetylases in gene regulation. Bioessays 20: 615–626 9780836 10.1002/(SICI)1521-1878(199808)20:8<615::AID-BIES4>3.0.CO;2-H

[bib26] Lindemann RK, Gabrielli B, Johnstone RW (2004) Histone-deacetylase inhibitors for the treatment of cancer. Cell Cycle 3: 779–788 15153801

[bib27] Lotan Y, Xu XC, Shalev M, Lotan R, Williams R, Wheeler TM, Thompson TC, Kadmon D (2000) Differential expression of nuclear retinoid receptors in normal and malignant prostates. J Clin Oncol 18: 116–121 10623701 10.1200/JCO.2000.18.1.116

[bib28] Macaluso M, Giordano A (2004) How does DNA methylation mark the fate of cells? Tumori 90: 367–372 15510977 10.1177/030089160409000401

[bib29] Mareel M, Leroy A (2003) Clinical, cellular, and molecular aspects of cancer invasion. Physiol Rev 83: 337–376 12663862 10.1152/physrev.00024.2002

[bib30] Marks PA, Richon VM, Rifkind RA (2000) Histone deacetylase inhibitors: inducers of differentiation or apoptosis of transformed cells. J Natl Cancer Inst 92: 1210–1216 10922406 10.1093/jnci/92.15.1210

[bib31] Miller TA, Witter DJ, Belvedere S (2003) Histone deacetylase inhibitors. J Med Chem 46: 5097–5116 14613312 10.1021/jm0303094

[bib32] Mueller PR, Coleman TR, Kumagai A, Dunphy WG (1995) Myt1: a membrane-associated inhibitory kinase that phosphorylates Cdc2 on both threonine-14 and tyrosine-15. Science 270: 86–90 7569953 10.1126/science.270.5233.86

[bib33] Nakayama T, Watanabe M, Yamanaka M, Hirokawa Y, Suzuki H, Ito H, Yatani R, Shiraishi T (2001) The role of epigenetic modifications in retinoic acid receptor beta2 gene expression in human prostate cancers. Lab Invest 81: 1049–1057 11454993 10.1038/labinvest.3780316

[bib34] Njar VC, Gediya L, Purushottamachar P, Chopra P, Belosay A, Patel JB (2006a) Retinoids in clinical use. Med Chem 2: 431–438 16848757 10.2174/157340606777724022

[bib35] Njar VC, Gediya L, Purushottamachar P, Chopra P, Vasaitis TS, Khandelwal A, Mehta J, Huynh C, Belosay A, Patel J (2006b) Retinoic acid metabolism blocking agents (RAMBAs) for treatment of cancer and dermatological diseases. Bioorg Med Chem 14: 4323–4340 16530416 10.1016/j.bmc.2006.02.041

[bib36] Ocker M, Schneider-Stock R (2007) Histone deacetylase inhibitors: signalling towards p21cip1/waf1. Int J Biochem Cell Biol 39: 1367–1374 17412634 10.1016/j.biocel.2007.03.001

[bib37] Patel JB, Huynh CK, Handratta VD, Gediya LK, Brodie AM, Goloubeva OG, Clement OO, Nanne IP, Soprano DR, Njar VC (2004) Novel retinoic acid metabolism blocking agents endowed with multiple biological activities are efficient growth inhibitors of human breast and prostate cancer cells *in vitro* and a human breast tumor xenograft in nude mice. J Med Chem 47: 6716–6729 15615521 10.1021/jm0401457

[bib38] Patel JB, Khandelwal A, Chopra P, Handratta VD, Njar VC (2007a) Murine toxicology and pharmacokinetics of novel retinoic acid metabolism blocking agents. Cancer Chemother Pharmacol 60(6): 899–905 17345084 10.1007/s00280-007-0438-3

[bib39] Patel JB, Mehta J, Belosay A, Sabnis G, Khandelwal A, Brodie AM, Soprano DR, Njar VC (2007b) Novel retinoic acid metabolism blocking agents have potent inhibitory activities on human breast cancer cells and tumour growth. Br J Cancer 96: 1204–1215 17387344 10.1038/sj.bjc.6603705 PMC2360155

[bib40] Patra SK, Patra A, Dahiya R (2001) Histone deacetylase and DNA methyltransferase in human prostate cancer. Biochem Biophys Res Commun 287: 705–713 11563853 10.1006/bbrc.2001.5639

[bib41] Phatak P, Cookson JC, Dai F, Smith V, Gartenhaus RB, Stevens MF, Burger AM (2007) Telomere uncapping by the G-quadruplex ligand RHPS4 inhibits clonogenic tumour cell growth *in vitro* and *in vivo* consistent with a cancer stem cell targeting mechanism. Br J Cancer 96: 1223–1233 17406367 10.1038/sj.bjc.6603691 PMC2360152

[bib42] Pili R, Kruszewski MP, Hager BW, Lantz J, Carducci MA (2001) Combination of phenylbutyrate and 13-*cis* retinoic acid inhibits prostate tumor growth and angiogenesis. Cancer Res 61: 1477–1485 11245454

[bib43] Qian DZ, Ren M, Wei Y, Wang X, van de Geijn F, Rasmussen C, Nakanishi O, Sacchi N, Pili R (2005) *In vivo* imaging of retinoic acid receptor beta2 transcriptional activation by the histone deacetylase inhibitor MS-275 in retinoid-resistant prostate cancer cells. Prostate 64: 20–28 15651062 10.1002/pros.20209

[bib44] Qian DZ, Wei YF, Wang X, Kato Y, Cheng L, Pili R (2007) Antitumor activity of the histone deacetylase inhibitor MS-275 in prostate cancer models. Prostate 67: 1182–1193 17520666 10.1002/pros.20611

[bib45] Richon VM, Sandhoff TW, Rifkind RA, Marks PA (2000) Histone deacetylase inhibitor selectively induces p21WAF1 expression and gene-associated histone acetylation. Proc Natl Acad Sci USA 97: 10014–10019 10954755 10.1073/pnas.180316197 PMC27656

[bib46] Robertson KD, Jones PA (2000) DNA methylation: past, present and future directions. Carcinogenesis 21: 461–467 10688866 10.1093/carcin/21.3.461

[bib47] Sonneveld E, van den Brink CE, van der Leede BM, Schulkes RK, Petkovich M, van der Burg B, van der Saag PT (1998) Human retinoic acid (RA) 4-hydroxylase (CYP26) is highly specific for all-trans-RA and can be induced through RA receptors in human breast and colon carcinoma cells. Cell Growth Differ 9: 629–637 9716180

[bib48] Verheul HM, Qian DZ, Carducci MA, Pili R (2007) Sequence-dependent antitumor effects of differentiation agents in combination with cell cycle-dependent cytotoxic drugs. Cancer Chemother Pharmacol 60: 329–339 17256134 10.1007/s00280-006-0379-2

[bib49] Villani MG, Appierto V, Cavadini E, Bettiga A, Prinetti A, Clagett-Dame M, Curley RW, Formelli F (2006) 4-oxo-fenretinide, a recently identified fenretinide metabolite, induces marked G2-M cell cycle arrest and apoptosis in fenretinide-sensitive and fenretinide-resistant cell lines. Cancer Res 66: 3238–3247 16540676 10.1158/0008-5472.CAN-05-3362

[bib50] Weinberg RA (2007) The Biology of Cancer. New York: Garland Science

